# Least-squares community extraction in feature-rich networks using similarity data

**DOI:** 10.1371/journal.pone.0254377

**Published:** 2021-07-15

**Authors:** Soroosh Shalileh, Boris Mirkin

**Affiliations:** 1 Department of Data Analysis and Artificial Intelligence, HSE University, Moscow, Russian Federation; 2 Laboratory of Methods for Big Data Analysis, HSE University, Moscow, Russian Federation; 3 Department of Computer Science and Information Systems, Birkbeck University of London, London, United Kingdom; University of Burgundy, FRANCE

## Abstract

We explore a doubly-greedy approach to the issue of community detection in feature-rich networks. According to this approach, both the network and feature data are straightforwardly recovered from the underlying unknown non-overlapping communities, supplied with a center in the feature space and intensity weight(s) over the network each. Our least-squares additive criterion allows us to search for communities one-by-one and to find each community by adding entities one by one. A focus of this paper is that the feature-space data part is converted into a similarity matrix format. The similarity/link values can be used in either of two modes: (a) as measured in the same scale so that one may can meaningfully compare and sum similarity values across the entire similarity matrix (summability mode), and (b) similarity values in one column should not be compared with the values in other columns (nonsummability mode). The two input matrices and two modes lead us to developing four different Iterative Community Extraction from Similarity data (ICESi) algorithms, which determine the number of communities automatically. Our experiments at real-world and synthetic datasets show that these algorithms are valid and competitive.

## 1 Introduction: Background, previous work, our approach

### 1.1 Background

Community detection is a popular research subject. Originally, this concerned “pure” network data. Then data of a set of features at the network nodes have been added to the between-node link weights, to refer to such two-fold data structures as “feature-rich” networks [1] or “node-attributed” graphs [2].

A community is a group of relatively densely inter-connected nodes that are similar in the feature space too. In the past decade, a number of papers with various approaches to identifying communities in feature-rich networks have been published. To classify them, we follow [3] to divide community detection methods according to the stage of the process of finding communities at which the two data types, network and features, are merged together. This may occur before the process begins (early fusion), within the process (simultaneous fusion), and after the process (late fusion).

Obviously, early fusion and late fusion approaches must be purely heuristic because they have nothing to do with modelling the observed data. The subject of our interest, methods based on data modelling, therefore lie within the simultaneous fusion stage. Among the data modelling approaches, we distinguish between theory-driven and data-driven approaches. Theory-driven approaches involve a model of the world leading to a probabilistic distribution, parameters of which can be recovered from the data. Data-driven approaches involve no world models but rather focus on modelling the data as is. According to this approach, the data is considered as an array of numbers to be recovered in the process of decoding a model that “encodes” the data. This view has been formulated at earlier stages of the history of statistical thinking. Popular data analysis methods—K-means clustering and Principal Component Analysis—naturally fall within this approach [4].

### 1.2 Previous work

Two data types, network and features, can be merged together in the process of finding communities before the process begins (early fusion), within the process (simultaneous fusion), and after the process (late fusion) [3]. Within the simultaneous fusion approach literature we distinguish data-driven modelling approaches—the niche in which this paper belongs. Two other approaches here are: theory-driven modelling and heuristics, which is the most numerous.

Among heuristic approaches to community detection in feature-rich networks, several adapt criteria of the classical clustering algorithms to the presence of two data sources. These classical clustering methods are: normalized cut and related spectral clustering [5], as well as the modularity-based method [6, 7] and Louvain algorithm [8] to detect communities by locally maximizing the modularity score. Paper [9] modifies the normalized cut criterion by adding the so-called unimodality compactness to reflect the homogeneity of attributes within a community. A modified modularity criterion and corresponding method is developed in [10]. A modified Louvain method is proposed and tested in [11]. The so-called network embedding (see [12–14]) is another popular heuristics development. In this, both the network and feature data are approximated with a low-dimensional Euclidean vector space.

Methods in [15–17] are based on the co-called Graph-Neural Networks (GNNs). To be more specific, in [15] an objective function is formulated as a continuous relaxation of the normalized cut problem and then a GNN is trained to compute cluster assignments minimizing this function. Paper [16] can be considered as a modified version of the popular Graph Convolutional Networks (GCNs) [18] for the task of clustering in feature-rich networks using the modularity criterion [6]. Paper [17] first combines the attribute and network data to apply then an autoencoding scheme for clustering in the space of thus obtained latent variables.

The theory-driven approach involves both the maximum likelihood and Bayesian criteria for fitting probabilistic models. A prominent concept here is stochastic block model (SBM), also inherited from the analysis of conventional networks. In [19] network structures are modeled with SBM while the continuous features are modeled with a Gaussian mixture model. The Blockmodel Entropy Significance Test (BESTest) [20] for evaluation of how much a metadata partition is relevant to the network structure. The BESTest works by first dividing network’s nodes according to the feature labels and then by computing the entropy of that SBM which best corresponds to the partition. In [21] a Cluster Representative SBM model is proposed, such that, instead of measuring the distance between node attributes in feature space, the distance between each node attributes and clusters representative prototypes is measured and then the SBM is modified correspondingly.

Methods in [22–24] are based on Bayesian inferences. In [25] the authors propose clustering criterion to statistically model interrelations between the network structure and node attributes.

The data-driven modeling approach seems somewhat less developed at this stage. Some authors propose the so-called non-negative matrix factorization (NNMF) to approximate the data via factorization of them in the product of non-negative matrices of simpler structure. In papers [26, 27] combined criteria for such an approximation and methods for suboptimally solving them are proposed. The criteria are based on the least-squares approach like that by ourselves. However, these criteria involve some derived data rather than the original ones. A different tackle is undertaken in [2]. Here, the data are summarized as given; the quality, however is scored according to the principle of minimum description length (MDL) so that the number of bits in coding of the summary is minimized. In [28] semidefinite programming (SDP) method is utilized to detect the communities. And moreover, a sparse attribute self-adjustment mechanism is introduced to determine the relative importance of each source of information, i.e node attributes or network links.

This paper combines aspects of the two approaches above: a straightforward modeling of the data as is, like in [2], and a least-squares criterion, like in [26, 27].

### 1.3 Our approach

We follow a conventional assumption that there is a hidden partition of the node set in non-overlapping communities, which is supplied with hidden parameters encoding the average link intensities in the network and similarity intensities in the similarities (i.e in the similarity space, obtained from the feature space, as explained in forthcoming subsection 2.1). These are used at the decoding stage so that the residuals of data recovery equations are minimized according to the least squares criterion. Such an approach is referred to as the data recovery approach in [4]; in neural network domain, that is referred to as auto-encoder [29].

The least squares criterion in this case leads to computationally hard problems which are usually tackled with various heuristics. In particular, we follow a greedy-wise strategy of sequentially extracting clusters one-by-one. This strategy has been applied earlier to either only similarity/network data [30, 31] or to only attribute/feature data [32, 33]. These authors applied this strategy to the case of combined feature and network data in a short note [34].

More precisely, we consider a network with features at the nodes, *A* = {*P*, *Y*}. Here *P* = (*p*_*ij*_) is an *N* × *N* matrix of mutual link scores between nodes *i*, *j* ∈ *I* where *I* is an *N*-element set of the network nodes; *Y* = (*y*_*iv*_) is a *N* × *V* matrix of feature values *y*_*iv*_ at nodes *i* = 1, 2, …, *N* with feature labels *v* = 1, 2, …, *V*. A flat network, at which inter-node links either exist or not, can be equivalently represented by *P* with 1/0 entries, so that *p*_*ij*_ = 1 if there is a link between *i* and *j*, and *p*_*ij*_ = 0, otherwise.

A community *S* ⊂ *I* is represented by its binary *N* × 1 indicator column-vector *s* = (*s*_*i*_) so that *s*_*i*_ = 1 if *i* ∈ *S*, and *s*_*i*_ = 0, otherwise. To adjust this to the network link scoring, we assign *S* with an intensity λ to be determined later. To express the idea that members of the community, ideally, share the same feature values, we assign *S* with its standard *V*-dimensional point *c* = (*c*_*v*_).

We assume that there is a partition **S** = {*S*_1_, *S*_2_, …, *S*_*K*_} of *I* in *K* non-overlapping communities, a.k.a. clusters, related to this dataset as described below [34].

Denote *k-th* *N*-dimensional binary cluster membership vector by *s*_*k*_ = (*s*_*ik*_); *s*_*ik*_ = 1 for *i* ∈ *I* being a member of the cluster, *s*_*ik*_ = 0, otherwise. The cluster is assigned with a *V*-dimensional center vector *c*_*k*_ = (*c*_*kv*_). Also, there is a positive network intensity weight of *k-th* cluster denoted by λ_*k*_. Here *k* is an index, *k* = 1, 2, …, *K*.

Our model requires that the data can be recovered from these according to equations, for network data,

$$\begin{array}{c} p_{ij}=\sum_{k=1}^{K}\lambda_{k}s_{ik}s_{jk}+e_{ij},i,j\in I, \end{array}$$ (1)

and for feature data,

$$\begin{array}{c} y_{iv}=\sum_{k=1}^{K}c_{kv}s_{ik}+f_{iv},i\in I,v\in V. \end{array}$$ (2)

where items *e*_*ij*_ and *f*_*iv*_ are residuals to be made as small as possible according to the least-squares criterion

$$\begin{array}{c} \begin{array}{c} F\left(\lambda_{k},s_{k},c_{k}\right)=\rho \sum_{k=1}^{K}\sum_{i,v}{\left(y_{iv}-c_{kv}s_{ik}\right)}^{2}\\ +\xi \sum_{k=1}^{K}\sum_{i,j}{\left(p_{ij}-\lambda_{k}s_{ik}s_{jk}\right)}^{2}. \end{array} \end{array}$$ (3)

which is to be minimized with respect to unknown membership vectors *s*_*k*_, community centers *c*_*k*_ and intensity weights λ_*k*_.

The factors *ρ* and *ξ* in Eq (3) are expert-driven constants to balance the relative weights between the two sources of data, network and features.

The operations of summation in criterion in Eq (3) are outside of the parentheses, whereas the models (1) and (2) require them to be within the parentheses. However, the formulation in (3) is consistent with the models in (1) and (2) because vectors *s*_*k*_ (*k* = 1, 2, …, *K*) correspond to a partition; thus, they are are mutually orthogonal. Therefore, for any specific *i*, *s*_*ik*_ is zero for all *k* except one, so that each of the sums over *k* in Eqs (1) and (2) consists of just one item, and the summation sign may be applied outside of the parentheses indeed.

The authors follow a doubly-greedy strategy for fitting the model in Eqs (1), (2), and (3) [34]. This strategy is based on a greedily finding only one cluster *T* = *S*_*k*_ at a time by minimizing that part of criterion in (3) related to *T* = *S*_*k*_ only:

$$\begin{array}{c} \begin{array}{c} F\left(\lambda ,t,c\right)=\rho \sum_{i,v}{\left(y_{iv}-c_{v}t_{i}\right)}^{2}+\xi \sum_{i,j}{\left(p_{ij}-\lambda t_{i}t_{j}\right)}^{2}. \end{array} \end{array}$$ (4)

with respect to unknown community *T* 1/0 membership vector *t* = (*t*_*i*_), community center *c* = (*c*_*v*_) and intensity weight λ.

The optimal community center *c* and intensity weight λ can be expressed through the data and membership vector *t* by using the first-order optimality conditions. Conveniently, the optimal *c* is the within-*T* mean of the vectors *y*_*i*_ over *i* ∈ *T* and the optimal λ is the average link score within *T*. By putting these expressions for *c* and λ into criterion (4) and making elementary algebraic transformations, we can reformulate (4) as

$$\begin{array}{c} F=\rho T(Y)+\xi T(P)-G(t) \end{array}$$ (5)

where *T*(*Y*) = ∑_*i*,*v*_ *y*_*iv*_^2^ is the quadratic feature data scatter, $T\left(P\right)=\sum_{ij}p_{i,j}^{2}$, the quadratic network data scatter, and

$$\begin{array}{c} G\left(t\right)=\rho |T|\sum_{v}c_{v}^{2}+\xi \lambda \sum_{i,j}p_{ij}t_{i}t_{j}=\rho |T|\sum_{v}c_{v}^{2}+\xi \lambda^{2}{\left|T\right|}^{2}. \end{array}$$ (6)

Therefore, to minimize the criterion (4), one may maximize criterion (6). This is where the other greedy part works in our approach. Specifically, we grow a suboptimal *T* from a singleton by adding nodes one by one to maximize the increment of *G*(*t*) at each step. An exact formulation of this algorithm, SEFNAC, will be given further on. Our experiments at real-world and synthetic datasets have shown that this approach is valid and competitive against existing state-of-the-art approaches [34].

In this paper we build over the approach proposed in [34] by extending that in two directions: (i) Converting feature data to a similarity matrix format, (ii) Taking into account two modes, summability and nonsummability, for using similarity data, as well as experimentally validating emerging algorithmic options.

Direction (i), Converting features into a similarity matrix format, is rather popular in data science, first of all, with regard to the so-called kernel functions. A kernel function *K*(*x*, *y*) models inner product between images of vectors *x* and *y* under a not necessarily linear mapping *w*, *w*(*x*) and *w*(*y*), in the so-called straightening space in which *K*(*x*, *y*) =< *w*(*x*), *w*(*y*) >. What is nice about it—in many cases, there is no need in using the transformation *w*(*x*) itself—the kernel *K*(*x*, *y*) suffices. One popular example of kernel function is the so-called Gaussian kernel defined as *K*(*x*, *y*) = *exp*(−*d*(*x*, *y*)/*α*) where *d*(*x*, *y*) =< *x* − *y*, *x* − *y* > is the squared Euclidean distance and *α* > 0, a normalising constant. Application of kernel functions usually is justified by the need to do intricate non-linear transformations of the feature space in situations at which the hidden interclass boundaries are curled and twisted. We limit ourselves with a simplest kernel function *F*(*x*, *y*) = < *x*, *y*>, the inner product, because we do not expect complicated shapes neither in the real world datasets under consideration, nor in the generated “synthetic” datasets because of rather simple data generation models.

Another consideration which influenced our choice is the so-called curse of dimensionality which is associated with the fact that the Euclidean distance in a high-dimensional feature space gets less informative of the mutual location of objects in the feature space, whereas the inner product perhaps is more steady of the angular information.

Therefore, we are going to consider *N* × *N* matrix *R* = *YY*^*T*^ to represent the feature data rather than the original *N* × *V* matrix *Y*.

Regarding direction (ii), Two modes of usage of similarity data, (a) Summability and (b) Nonsummability, we mean the following. In the Summability mode, we consider all link scores as measured in the same scale, so that it makes sense to sum them across the entire table or any part of it. The Nonsummability mode relates to the case at which each node’s links are considered as scored in different scales. In this case, it makes sense to sum link scores only within a row or column, but not across different rows.

The Nonsummability assumption may have sense, for example, in some psychological experiments in which the entities are individuals or cognitive subsystems with different scales of individual judgements. Another example: two sets of internet sites; one to provide classical music education, the other to sell goods. These sets would much differ in the following: (a) the numbers of visitors: they are massive at selling goods sites, and they are much more modest at classical music education sites; (b) the time spent: that would be of the order of seconds at purchasing goods and hours at listening music.

Each of the two modes can be considered at each of the similarity data matrices, *P* and *R* = *YY*^*T*^, which generates four different cases. We apply the least squares approach to all the cases and conduct a comprehensive set of experiments to validate and to compare the performance of the newly proposed algorithms.

Our experiments show that this approach is able to recover hidden clusters in feature-rich networks using similarity data indeed. Moreover, it appears the conversion of feature data to similarity data in some cases leads to more accurate cluster recovery results in comparison to the use of the original *Y* dataset. Overall, our experiments show that the proposed methods are competitive against other state-of-the-art approaches.

## 2 Methodology

### 2.1 Inner products as similarites

Our approach assumes a preliminary standardization of the data, both the network and feature spaces. The features are standardized by subtracting the means *g*_*v*_ = ∑_*i*∈*I*_ *y*_*iv*_/*N* from feature columns *v*, *v* = 1, 2, …, *V*. To distinguish *g*_*v*_ from within-cluster means, they frequently are referred to as grand means. We accept the row-to-row inner product *r*_*ij*_ = < *y*_*i*_, *y*_*j*_ > = ∑_*v*∈*V*_ *y*_*iv*_ *y*_*jv*_ as the similarity index. Each feature *v* contributes the product *y*_*iv*_ *y*_*jv*_ to this, which much depends on the mutual location of *i* and *j* nodes on the axis *v* with respect to the grand mean *g*_*v*_.

The product is positive when both node location are either larger than *g*_*v*_ = 0 or smaller than *g*_*v*_ = 0. It is negative when *i* and *j* are on different sides from *g*_*v*_ = 0. Furthermore, the closer *y*_*iv*_ and *y*_*jv*_ to zero the smaller the product and the farther they are from zero, the greater the product.

Scoring the similarity by the inner product makes those entities in which features are further away from the grand mean, more distinguishable. In contrast, those entities in which feature values are close to the grand mean are less distinguishable, therefore they might be merged during the clustering process.

### 2.2 Data recovery models for a single community detection in a similarity matrix

As explained above, we have two *N* × *N* data matrices, matrix *R* = (*r*_*ij*_) of feature-based node-to-node similarities and matrix *P* = (*p*_*ij*_) of node-to-node link scoring. To unify our presentation, we are going to denote either of them as *B* = (*b*_*ij*_) where *b*_*ij*_ stands for either a converted feature-based similarity *r*_*ij*_ or a ‘native’ link weight *p*_*ij*_ (*i*, *j* ∈ *I*).

To define our data-driven community model, let us specify the following notation.

A community, or cluster, *T* ⊂ *I* is represented by a binary *N* × 1 membership column vector, *t* = (*t*_*i*_) in which *t*_*i*_ = 1 if *i* ∈ *T*, and *t*_*i*_ = 0, otherwise.

Assume that there may be two possible modes of using the similarity scores *b*_*ij*_:

SM Summability Mode
  In this mode, the similarities *b*_*ij*_ are comparable and summable across the entire matrix *B*. In this case, there should an intensity value *η* to relate the similarity measurement scale to *T*. Specifically, each within-community similarity *b*_*ij*_ *i*, *j* ∈ *T*, should be approximately equal to the intensity *η* for *T*.
NM Nonsummability Mode
  In this mode, the similarities *b*_*ij*_ in any column *j* are assumed to be non-comparable to similarities *b*_*ij*′_ in any different column *j*′ ≠ *j*, *i*, *j* ∈ *I*. Therefore, a specific intensity *η*_*j*_ is assumed for each column *j* ∈ *I*, so that, for any *i* ∈ *T* the similarity value *b*_*ij*_ should approximate the value *η*_*j*_.

The SM mode is typical in network analysis. NM mode points to not an uncommon data type emerging in some psychological experiments in which the nodes are individuals or cognitive subsystems with different scales of individual judgements. Similarly, between-industries input-output tables in Economics may use different measurement scales for production of different industries, especially for raw materials such as electricity, coal, and oil. The similarity data derived from the feature tables also may be considered as measured in NM mode sometimes, especially in potentially important situations at which some nodes *j* may be considered as more important than the other—then similarity to each of them could serve as that measured in a different scale.

To relate a community *T* to the similarity data *B*, we assume that a unified intensity *η* exists in the SM mode or a set of intensity values *η*_*j*_, *j* ∈ *I*, in the NM mode, so that either of the two following approximate equations holds:

$$\begin{array}{c} b_{ij}=\eta s_{i}s_{j}+e_{ij},i,j\in I, \end{array}$$ (7)

at the SM, or

$$\begin{array}{c} b_{ij}=\eta_{j}s_{i}+e_{ij},i,j\in I. \end{array}$$ (8)

at the NM assumption.

Since there are two sources of data, namely, the feature-based similarity data and the network data, and for each of them either of the two modes can be accepted, consequently, there will be four possible combinations of modes and data sources. As a convention, we assume that symbol “S” stands for the summability mode, and “N” stands for the nonsummability mode, at each of the data sources, so that the first letter refers to the feature-based similarity data, whereas the second letter refers to the network data. Consequently, combination SS refers to the case at which both data sets are in the Summable mode; SN, to the case at which the feature-based similarity data are Summable and the network data are not; NS, to the case at which the feature-based similarity data are Nonsummable and the network data are Summable; NN, to the case at which both data sets are in the Nonsummable mode. To avoid repetitive derivations, we consider in detail only one of the four cases, say, SN.

By using the least-squares approach, we arrive at the problem of finding a hidden membership matrix *s* = (*s*_*ik*_), intensities for the similarity data *μ*_*k*_ and intensity weights λ_*jk*_ minimizing the sum of squared residuals according to the SN mode:

at SN assumption:

$$\begin{array}{c} \begin{array}{c} F_{SN}\left(s_{k},\mu_{k},\lambda_{jk}\right)=\rho \sum_{k=1}^{K}\sum_{i,j}{\left(r_{ij}-\mu_{k}s_{ik}s_{jk}\right)}^{2}\\ +\xi \sum_{k=1}^{K}\sum_{i,j}{\left(p_{ij}-\lambda_{jk}s_{ik}\right)}^{2}, \end{array} \end{array}$$ (9)

The factors *ρ* and *ξ* in Eq (9) are expert-driven constants to balance the relative weights of the two sources of data, network links and feature-based similarity values. In this paper, they are taken to be equal to unity each.

Since vectors *s*_*k*_ = (*s*_*ik*_) (*k* = 1, 2, …, *K*) correspond to a partition, they are mutually orthogonal. That means that for any specific *i*, *s*_*ik*_ is zero for all *k*’s except one: that one *k* for which *S*_*k*_ contains *i*. As a result, each of the sums over *k* in the models relates to a single summand, meaning that the operation of summation over *k* may be applied outside of the parentheses in Eq (9).

### 2.3 The iterative community extraction with least-squares

Global optimization of the criterion (9) is computationally expensive and cannot be achieved in a reasonable time. Therefore, there can be various heuristic strategies applied. We are going to exploit a doubly greedy approach of sequential extraction [35]. This approach can be applied here because the criteria to optimize are additive. According to this approach, parts *S*_*k*_ of the partition *S* are sought not simultaneously, but one-by-one, sequentially, in a greedy manner. That is, a subset of *I* to serve as *S*_*k*_ at *k* = 1 is found to minimize the part of the criterion related to *S*_1_.

Specifically, for an individual community denoted by *T* ⊆ *I*, its membership by *t* = (*t*_*i*_), so that *t*_*i*_ = 1 if *i* ∈ *T* and *t*_*i*_ = 0, otherwise; its intensity similarity by *μ*; and the corresponding intensity weight by λ_*j*_ (the index *k* has been removed), the extent of fit between the community and the dataset, according to criterion (9), is

$$\begin{array}{c} \begin{array}{c} f_{SN}\left(\mu ,\lambda_{j},t_{i}\right)=\rho \sum_{i,j}{\left(r_{ij}-\mu t_{i}t_{j}\right)}^{2}+\;\\ \xi \sum_{i,j}{\left(p_{ij}-\lambda_{j}t_{i}\right)}^{2} \end{array} \end{array}$$ (10)

We take a subset *T* minimizing, in some sense, the criterion (10) as the first part of partition *S* we are to find, *S*_1_. Then this *S*_1_ is removed from *I* and the next part, *S*_2_, is sought in the same way over the residual entity set *I*′ ← *I* − *S*_1_. This continues till a pre-specified stopping criterion is reached such as, say, that the residual *I*′ gets empty.

Within this greedy strategy, at its *k*-th step (*k* = 1, 2, …, *K*), we use one more greedy procedure for obtaining a (locally) optimal set *T* and its quantitative characteristics *μ* and λ_*j*_, *j* ∈ *I*. The additive structure of the criterion (10) allows us to express them using contributions to the data scatter.

Consider two partial criteria, the two individual items in the squared error criterion (10):

(a) The fit between the summable community model and the similarity data:

$$\begin{array}{c} F_{RS}\left(\mu ,t\right)=\sum_{i,j}{\left(r_{ij}-\mu t_{i}t_{j}\right)}^{2} \end{array}$$ (11)

(b) The fit between the nonsummable community model and the network data:

$$\begin{array}{c} F_{PN}\left(\lambda ,t\right)=\sum_{i,j}{\left(p_{ij}-\lambda_{j}t_{i}\right)}^{2}. \end{array}$$ (12)

The total goodness of fit measure is *f*_*SN*_ = *ρF*_*RS*_ + *ξF*_*PN*_ where *ρ* and *ξ* are user-defined weights balancing two data sources, the feature-based similarities and the network links, respectively.

At a given *T* ⊆ *I*, to minimize the criterion (10) with respect to the quantitative characteristics *μ* and λ_*j*_, one should apply the first-order optimality conditions. The derivatives of *f*_*SN*_ over *μ* and λ_*j*_ are:

$$\begin{array}{c} \frac{\partial F_{RS}}{\partial \mu }=2\rho \sum_{i,j}\left(r_{ij}-\mu t_{i}t_{j}\right)\left(-t_{i}t_{j}\right). \end{array}$$ (13)

and

$$\begin{array}{c} \frac{\partial F_{PN}}{\partial \mu_{j}}=2\xi \sum_{i,j}\left(r_{ij}-\lambda_{j}t_{i}\right)\left(-t_{i}\right). \end{array}$$ (14)

Equating them to zero yields:

$$\begin{array}{c} \sum_{i,j}r_{ij}t_{i}t_{j}=\mu \sum_{i}t_{i}^{2}\sum_{j}t_{j}^{2}, \end{array}$$ (15)

and

$$\begin{array}{c} \sum_{i}p_{ij}t_{i}=\lambda_{j}\sum_{i}t_{i}^{2}. \end{array}$$ (16)

Since *t*_*i*_ is 1/0 binary, equality $t_{i}^{2}=t_{i}$ holds. Thus, $\sum_{i}t_{i}^{2}=\sum_{j}t_{j}^{2}=\sum_{i}t_{i}=\left|T\right|$. Therefore, these equations can be equivalently reformulated as follows:

$$\begin{array}{c} \mu =\frac{\sum_{i,j}r_{ij}t_{i}t_{j}}{{\left|T\right|}^{2}}=\frac{\sum_{i,j\in T}r_{ij}}{{\left|T\right|}^{2}}, \end{array}$$ (17)

and

$$\begin{array}{c} \lambda_{j}=\frac{\sum_{i}p_{ij}t_{i}}{\left|T\right|}=\frac{\sum_{i\in T}p_{ij}}{\left|T\right|}. \end{array}$$ (18)

In other words, the optimal *μ* and λ_*j*_ must be central in *T*: they are within-cluster means of the corresponding similarity and link scoring values.

Let us now reformulate the partial criteria (11) and (12) by opening the parentheses and putting there the found optimal values of *μ* and λ_*j*_:

Criterion (11) yields:

$$F_{RS}\left(\mu ,t\right)=\sum_{i,j}{\left(r_{ij}-\mu t_{i}t_{j}\right)}^{2}=\sum_{i,j}r_{ij}^{2}-2\mu \sum_{i,j}r_{ij}t_{i}t_{j}+\mu^{2}\sum_{i,j}t_{i}t_{j}.$$

Let us denote the square *R* matrix scatter by $Q\left(R\right)=\sum_{i,j}r_{ij}^{2}$ and take into account that ∑_*i*,*j*_ *r*_*ij*_ *t*_*i*_ *t*_*j*_ = *μ* ∑_*i*,*j*_ *t*_*i*_ *t*_*j*_. Then the equation above can be rewritten as

$$\begin{array}{c} F_{RS}\left(\mu ,t\right)=Q\left(R\right)-\mu^{2}{\left|T\right|}^{2} \end{array}$$ (19)

Similarly, criterion (12) yields:

$$\begin{array}{c} F_{PN}\left(\lambda_{j},t\right)=\sum_{i,j}{\left(p_{ij}-\lambda_{j}t_{i}\right)}^{2}=\sum_{i,j}p_{ij}^{2}-2\sum_{i,j}p_{ij}t_{i}\lambda_{j}+\sum_{j}\lambda_{j}^{2}\sum_{i}t_{i}. \end{array}$$

Let us take into account that ∑_*i*_ *p*_*ij*_ *t*_*i*_ = λ_*j*_ ∑_*i*_ *t*_*i*_. Then the equation above can be rewritten as

$$\begin{array}{c} F_{PN}\left(\lambda ,t\right)=Q\left(P\right)-\sum_{j}\lambda_{j}^{2}\left|T\right|. \end{array}$$ (20)

where $Q\left(P\right)=\sum_{i,j}p_{ij}^{2}$ is the data *P* scatter.

Therefore, with the optimal values for *μ*, and λ_*j*_, the criterion (10) can be equivalently reformulated as:

$$\begin{array}{c} f(\mu ,\lambda ,t)=\rho Q(R)+\xi Q(P)-G \end{array}$$ (21)

where

$$\begin{array}{c} G\left(T\right)=G_{SN}=\rho \mu \sum_{ij}r_{ij}t_{i}t_{j}+\xi |T|\sum_{j}\lambda_{j}^{2}={\rho |T|}^{2}\mu^{2}+\xi \left|T\right|\sum_{j}\lambda_{j}^{2} \end{array}$$ (22)

where $\mu =\frac{\sum_{i,j}r_{ij}t_{i}t_{j}}{\sum_{i,j}t_{i}t_{j}}$ and $\lambda_{j}=\frac{\sum_{i\in T}p_{ij}}{\left|T\right|}$.

Maximizing criterion *G*(*T*) in the Eq (22) is equivalent to minimizing the corresponding one-cluster least-squares criteria Eq (10). Therefore, it makes sense to take a look whether *G*(*T*) has any meaning of its own.

First of all, we can rewrite the Eq (21) as a Pythagorean decomposition of the combined data scatter *Q*(*R*, *P*) = *ρQ*(*R*) + *ξQ*(*P*):

$$\begin{array}{c} Q(R,P)=\rho Q(R)+\xi Q(P)=G+f \end{array}$$ (23)

in two parts, the minimized squared residuals *f* (10) and the complementary part *G*. The decomposition gives a statistical meaning to the value of *G*. This is contribution of the community *T* to the combined data scatter *Q*(*R*, *P*).

A more intuitive meaning of the criterion one can see in the formula (22): it requires maximizing the size |*T*| of the community to be found and, simultaneously, maximizing the average within-community similarity and the squared distance from the vector (λ_*j*_) to 0.

Assuming that the data matrices are pre-processed so that the origin is transferred to the center of gravity, or grand mean, the point whose components are the averages of the corresponding similarity/network values, we may conclude that the cluster *T* should be both numerous and anomalous.

We refer to our local search algorithm for maximizing criterion (22) as to the Least-Squares Community Extraction from Similarity data, LS CESi or just CESi when the least-squares framework is assumed undoubtedly. We add to this an ending, sn, to indicate in the modified abbreviation, CESisn, that the summability mode is accepted for the feature-based similarity, and the nonsummability mode is accepted for the network links, s for SM and n for NM, in the case under consideration. The other three combinations will be referred to as CESiss, CESins, and CESinn, to mean combinations SM and SM, NM and SM, and NM and NM, respectively.

The algorithm finds a cluster *T* and its intensities *μ* and λ_*j*_ by locally maximizing *G* in the system of neighborhoods defined by the the condition that *T*’s neighborhood consists of subsets differing from *T* by just adding a single entity.

The CESi algorithm starts from a random *i* ∈ *I*. This *i* serves as the seed forming a starting singleton cluster *T* = {*i*}. This triggers execution of the base CESi module. At any current *T*, this module computes increment Δ(*j*) = *G*(*T* + *j*) − *G*(*T*) for every element *j* ∈ *I* − *T* and selects that j* at which Δ(*j*) is maximum. If this maximum is positive, then j* is added to *T*, and the module runs again from thus updated *T*. If, in contrast, Δ(j*) < 0, the algorithm halts and outputs *T* and its intensities *μ* and λ_*j*_, as well as its contribution to the combined data scatter *G*. Then the last check is performed: **Seed Relevance Check**: If the removal of the seed increases the cluster contribution; this seed is extracted from the cluster.

The algorithm CESi serves as the core subroutine in our Iterative community detection algorithm ICESi.

The algorithm ICESi starts by standardizing the square *N* × *N* matrices *R* and *P*—this will be described later. Then we set *k* = 1 and *I*_*k*_ = *I*. At a given *k*, we apply CESi to *R* and *P* data matrices restricted to the set *I*_*k*_. The resulting cluster *T* forms next cluster *S*_*k*+1_ along with its intensities *μ*_*k*+1_ and λ_*j*,*k*+1_, as well as the relative contribution to the combined data scatter *q*_*k*+1_ = *G*/*Q*(*R*, *P*). Now we redefine *I*_*k*+1_ = *I*_*k*_ − *S*_*k*+1_ and test a pre-specified stop-condition. The stop-condition is a predicate that may involve several clauses. One of them is testing whether *I*_*k*+1_ = ∅ or not. Two other clauses usually are limits to the current and cumulative contributions. To stop, the former should be less than, say, 5% of the *Q*(*R*, *P*), whereas the latter should be 50% of that or greater. If the stop condition is satisfied, we define *K* = *k* + 1 and output the found clusters *S*_*k*_ together with their numerical characteristics (*k* = 1, 2, …, *K*). Otherwise, we update *k* by adding 1, *k* ← *k* + 1 and execute the next iteration of extracting clusters.

Algorithms ICESiss, ICESins, ICESinn corresponding to other combinations of summability modes also use the decomposition (21) to maximize the contribution *G*, that is expressed either as

$$\begin{array}{c} G\left(T\right)=G_{SS}=\rho \mu \sum_{ij}r_{ij}t_{i}t_{j}+\xi \lambda \sum_{ij}p_{ij}t_{i}t_{j} \end{array}$$ (24)

at the combination SM and SM, or as

$$\begin{array}{c} G\left(T\right)=G_{NS}=\rho \left|T\right|\sum_{j}\mu_{j}^{2}+\xi \lambda \sum_{ij}p_{ij}t_{i}t_{j} \end{array}$$ (25)

at the combination NM and SM, or as

$$\begin{array}{c} G\left(T\right)=G_{NN}=\left|T\right|\left(\rho \sum_{j}\mu_{j}^{2}+\xi \sum_{j}\lambda_{j}^{2}\right) \end{array}$$ (26)

at the combination NM and NM.

At the assumption NN, where *μ*, *μ*_*j*_, λ, and λ_*j*_ are the corresponding within-*T* means. The algorithm ICESi works with them similarly, up to obvious modifications of the increment Δ(*j*).

A Python source code of thus defined ICESi can be found at https://github.com/Sorooshi/ICESi.

## 3 Setting of experiments for validation and comparison of the proposed methods

To set a computational experiment, one should specify its constituents:

1. A set of algorithms under comparison.
2. A set of datasets at which the algorithms are evaluated and/or compared.
3. A set of pre-processing methods which are applied to standardize or to normalize the datasets.
4. A set of criteria for assessment of the experimental results.

We address these, in sequence, in separate sections.

### 3.1 Algorithms under comparison

We take for comparison two algorithms of the model-based approach, CESNA [25], SIAN [24] which have been extensively tested in computational experiments. Besides, author-made codes of the algorithms are publicly available. We add to this our method SEFNAC [34] extracting communities from the data without converting them to the similarity format. We also tested the algorithm PAICAN from [23] in our experiments. The results of this algorithm, unfortunately, were always less than satisfactory; therefore, we excluded the algorithm PAICAN from this paper.

Here are brief descriptions of the three competitors.

#### 3.1.1. CESNA [25] overview

Given an undirected graph *G*(*V*, *E*) with binary node attribute matrix *X*, where *V* is the set of vertices and *E* is the set of edges, the aim of CESNA is to detect *C* communities regarding the graph structure and node attributes. The authors define two generative models, one for the graph and the other for attributes, and combine them together. For graph structure they use Eq (27) to model the probability of an edge between two nodes *u* and *v* as follows:

$$\begin{array}{l} P_{uv}=1-exp(-\sum_{c=1}^{C}F_{uc}F_{vc})\\ A_{uv}\sim Bernoulli(P_{uv}) \end{array}$$ (27)

where *A* ∈ {0, 1}^*N*×*N*^ denotes the graph adjacency matrix. Unknown function *F*_*uc*_ represents the membership of node *u* to community *c*, so that the probability is a logistic function of the inner product of *F*_*uc*_ and *F*_*vc*_. The presence or absence of an edge *uv* is governed by a Bernoulli distribution, so that it holds with probability *P*_*uv*_ or does not, with probability 1 − *P*_*uv*_.

A similar model (28) is defined for any binary attribute at nodes:

$$\begin{array}{c} \begin{array}{c} Q_{uk}=\frac{1}{1+exp(-\sum_{c}W_{kc}.F_{uc})}\\ X_{uk}∼Bernoulli\left(Q_{uk}\right) \end{array} \end{array}$$ (28)

Here *W*_*kc*_ is a real-valued parameter of the logistic model for community *c* to the *k-th* node attribute.

With the two models above, the problem is to infer values of latent variables *F* and *W* by maximizing the likelihood *l*(*F*, *W*) = *logP*(*G*, *X*|*F*, *W*) of the observed data G, X. Here *F* = (*F*_*uc*_) is the node-to-community membership matrix and *W* = (*W*_*kc*_) is the real-valued logistic model parameter for attributes.

Assuming that these two sources of data are conditionally independent, the loglikelihood can be defined as log *P*(*G*, *X*|*F*, *W*) = *L*_*G*_ + *L*_*X*_ where *L*_*G*_ = log *P*(*G*|*F*) and *L*_*X*_ = log *P*(*X*|*F*, *W*). To find *F* and *W* maximizing *L*_*G*_ and *L*_*X*_, which can be computed using the Eqs (27) and (28), the authors adopt projected gradient ascent approach with backtracking line search [36].

An author-supplied code for CESNA algorithm can be found at [37].

#### 3.1.2 SIAN [24] overview

Consider a set of features **x** = {*x*_*u*_} at nodes *u* = 1, 2, …, *n* and a set of node degrees **d** = {*d*_*u*_}. Assume, first, that each node *u* belongs to community *s* with the probability depending on *x*_*u*_. and denote all possible combinations of features and communities by Γ = (*γ*_*sx*_). Then the full prior probability of community assignment is *P*(**s**|Γ, **x**). At the next stage, edges between nodes are formed independently at random, with the probability of an edge between nodes *u* and *v* being $p_{uv}=d_{u}d_{v}\theta_{s_{u}s_{v}}$. Here *θ*_*st*_ is a hyper-parameter.

The task is to fit the model to the observed data by using the maximum likelihood principle. To this end, a binary adjacency matrix **A** = (*a*_*uv*_), is generated according to the following model:

$$\begin{array}{l} P(\text{A}|\Theta ,\Gamma ,x)=\sum_{s}P(\text{A}|\Theta ,\text{s}).P(\text{s}|\Gamma ,\text{x})\\ \;=\sum_{s}\prod_{u<v}p_{uv}^{a_{uv}}(1-p_{uv}{)}^{1-a_{uv}}\prod_{u}\gamma_{s_{u},x_{u}} \end{array}$$ (29)

Here Θ is a *k* × *k* matrix of elements *θ*_*st*_, and the sum is over all admissible node-to-community assignments. To maximise the function in (29) the authors use the Expectation-Maximisation (EM) algorithm.

An author-supplied code for SIAN algorithm can be found at [38].

#### 3.1.3. SEFNAC [34] overview

Given an *N* × *V* entity-to-feature matrix *Y* = (*y*_*iv*_), and an *N* × *N* adjacency matrix *P* = (*p*_*ij*_), the task is to cluster nodes sharing similar feature values while being densely connected according to *P*. This task in [34] is formulated as of minimization of the criterion

$$\begin{array}{c} \begin{array}{c} F\left(\lambda_{k},s_{k},c_{k}\right)=\rho \sum_{k=1}^{K}\sum_{iv}{\left(y_{iv}-c_{kv}s_{ik}\right)}^{2}\\ +\xi \sum_{k=1}^{K}\sum_{ij}{\left(p_{ij}-\lambda_{k}s_{ik}s_{jk}\right)}^{2} \end{array} \end{array}$$ (30)

where *c*_*k*_ represents the centroid vector of *k*-th community, λ_*k*_ its link intensity, and *s*_*ik*_ is a binary 1/0 value representing the membership of the *i*-th node to the *k*-th community.

By applying the optimality conditions, SEFNAC is a method for one-by-one extracting communities similar to the method ICESi; more detail can be found in [34].

An author-supplied code for SEFNAC algorithm is available at [39].

### 3.2 Datasets

We use both real world datasets and synthetic datasets. We describe them in the following subsections.

#### 3.2.1 Real world datasets

Two of the algorithms under comparison, unlike SEFNAC and ICESi, restrict the features to be categorical. Therefore, whenever a data set contains a quantitative feature we convert that feature to a categorical version. A brief overview of the five real-world data sets under consideration can be found in Table 1.

**Table 1:** Real world datasets under consideration.

Name	Nodes	Edges	Features	Ground Truth
Malaria HVR6	307	6526	6	Cys Labels
Lawyers	71	339	18	Derived out of office and status features
World Trade	80	1000	16	Structural world system in 1980 features
Parliament	451	11646	108	Political parties
COSN	46	552	16	Region

Symbols N, E, and F stand for the number of nodes, the number of edges, and the number of node features, respectively.

Here are brief descriptions of them.

*3.2.1.1 Malaria data set*. This data set is introduced in [40]. The nodes are amino acid sequences containing six highly variable regions (HVR) each. The edges are drawn between sequences with similar HVRs 6. In this data set, there are two nominal attributes of nodes:

1. Cys labels derived from of a highly variable region HVR6 sequence
2. Cys-PoLV labels derived from the sequences adjacent to regions HVR 5 and 6

The Cys Labels is considered as the ground truth.

*3.2.1.2 Lawyers dataset*. The Lawyers dataset comes from a network study of corporate law partnership carried out in a Northeastern US corporate law firm, referred to as SG & R, 1988-1991, in New England. It was introduced in [41] and it is available for downloading at [42]. There is a friendship network between lawyers in the study. The features in this dataset are:

1. Status (partner, associate),
2. Gender (man, woman),
3. Office location (Boston, Hartford, Providence),
4. Years with the firm,
5. Age,
6. Practice (litigation, corporate),
7. Law school (Harvard or Yale, UCon., Other)

Most features are nominal. Two features, “Years with the firm” and “Age”, are quantitative. We use the nominal format by authors of the previous studies. The categories of “Years with the firm” are *x* <= 10, 10 < *x* < 20, and *x* >= 20; the categories of “Age” are *x* <= 40, 40 < *x* < 50, and *x* >= 50.

The combination of Office location and Status is considered as the ground truth. (see Table 2).

**Table 2:** Features in the Lawyers dataset.

No.	Feature	Type	Categories	N	E	F
1	Status	Nominal	Partner, Associate			
2	Gender	Nominal	Male, Female			
3	Office	Nominal	Boston, Hartford, Providence			
4	Years with Firm	Categorised	*x* <= 10, 10 < *x* < 20, *x* >= 20	71	399	18
5	Age	Categorised	*x* <= 40, 40 < *x* < 50, *x* >= 50			
6	Practice	Nominal	Litigation, Corporate			
7	Law School	Nominal	Harvard, Yale, UCon.			

Symbols N, E, and F denote the number of nodes, the number of edges, and the number of node features, respectively.

*3.2.1.3 World-Trade dataset*. The World-Trade dataset contains data on trade between 80 countries in 1994 (see [43]). The link scores represent total imports by row-countries from column-countries, in $ 1,000, for the class of commodities designated as ‘miscellaneous manufactures of metal’ to represent high technology products or heavy manufacture. The scores for imports with values less than 1% of the country’s total imports are zeroed.

The node attributes are:

1. Continent (Africa, Asia, Europe, North America, Oceania, South America)
2. Structural World System Position (Core, Semi-Periphery, Periphery),
3. Gross Domestic Product per capita in $ (GDP p/c)
4. Structural World System Position in 1980 according to Smith and White (Core, Semi-Periphery, Periphery, N.A.I)
  N.A.I: stands for Not Available information which represent countries in which due to War to dictatorships etc their ecumenical information were not available.

The Structural World System Position in 1980 according to Smith and White is considered as the ground truth.

The GDP p/c feature is converted into a three-category nominal feature manually, according to the minima at its histogram. The categories are defined as follows: ‘Poor’ category is for the GDP less than $4406:9; ‘Mid-Range’ category is for the GDP greater than than $4406:9 but not greater than $21574:5; and ‘Wealthy’ category corresponds to the GDP greater than $21574:5.

These features are reviewed in Table 3. Before applying SEFNAC, all attribute categories are converted into 0/1 dummy variables which are considered quantitative.

**Table 3:** Features in World Trade data set.

No.	Feature	Type	Categories	N	E	F
1	Continent	Nominal	Africa, Asia, Europe, North America, Oceania, South America			
2	SWSP in 1994	Nominal	Core, Semi-periphery, Periphery	80	1000	16
3	GDP categories	Nominal	Poor, Mid-Range, Wealthy			
4	SWSP in 1980 according Smith and White	Nominal	Core, Semi-periphery, Periphery, N.I.A			

SWSP stands for Structural World System Position; GDP, for Gross Domestic Product per capita. Symbols N, E, and F show the number of nodes, the number of edges, and the number of node features, respectively.

*3.2.1.4 Parliament dataset*. In the Parliament data set, introduced in [23], nodes correspond to members of the French Parliament. An edge is drawn if the corresponding MPs have signed a bill together. The features are the constituency of MPs and their political party, as it is described by the authors. The latter is considered the ground truth (see Table 4).

**Table 4:** The Parliament data set.

No.	Feature	Type	Categories	N	E	F
1	Constituency	Nominal	MPs constituency	451	11464	108

Symbols N, E, and F show the number of nodes, the number of edges, and the number of node features, respectively.

*3.2.1.5 Consulting Organisational Social Network (COSN) dataset*. The Consulting Organisational Social Network (COSN) dataset is introduced in [44]. Nodes in this network correspond to employees in a consulting company. The (asymmetric) edges are formed in accordance with their replies to this question: “Please indicate how often you have turned to this person for information or advice on work-related topics in the past three months”. The answers are coded by 0 (I Do Not Know This Person), 1 (Never), 2 (Seldom), 3 (Sometimes), 4 (Often), and 5 (Very Often). Either of these 6 numerals is the weight of all the corresponding edges.

Nodes in this network have the following attributes:

1. Organisational level (Research Assistant, Junior Consultant, Senior Consultant, Managing Consultant, Partner),
2. Gender (Male, Female),
3. Region (Europe, USA),
4. Location (Boston, London, Paris, Rome, Madrid, Oslo, Copenhagen).

The Region feature is considered as the ground truth. A description of the data is in Table 5.

**Table 5:** The Consulting Organisational Social Network data set.

No.	Feature	Type	Categories	N	E	F
1	Organisational Level	Nominal	Assistant, Junior, Consultant, Senior Consultant, Managing Consultant, Partner	46	552	16
2	Gender	Nominal	Male, Female			
3	Region	Nominal	Europe, USA,			
4	Location	Nominal	Boston, London, Paris, Rome, Madrid, Oslo, Copenhagen			

N, E, and F show the number of nodes, the number of edges, and the number of node features, respectively.

#### 3.2.2 Generating synthetic datasets

In this section, we describe how we generate synthetic datasets with an innate cluster structure by separately generating:

- network;
- categorical features;
- quantitative features.

Each of these is put in a separate subsection.

*3.2.2.1 Generating network*. First, the number of nodes, *N*, and the number of communities *K* are specified. Then cardinalities/sizes of communities are defined randomly, up to a constraint that no community has less than a pre-specified number of nodes (in our experiments, this is set to 30, so that probabilistic approaches are applicable), and the total number of nodes in all the communities sums to *N*. We consider two settings for *N*: (a) *N* = 200, at a small size network, and (b) *N* = 1000, for a medium-size network. We postpone analysis of larger networks for another paper.

Given the community sizes, we populate them with nodes, that are specified just by indices. Then we specify two probability values, *p* and *q*.

**Fig 1:**
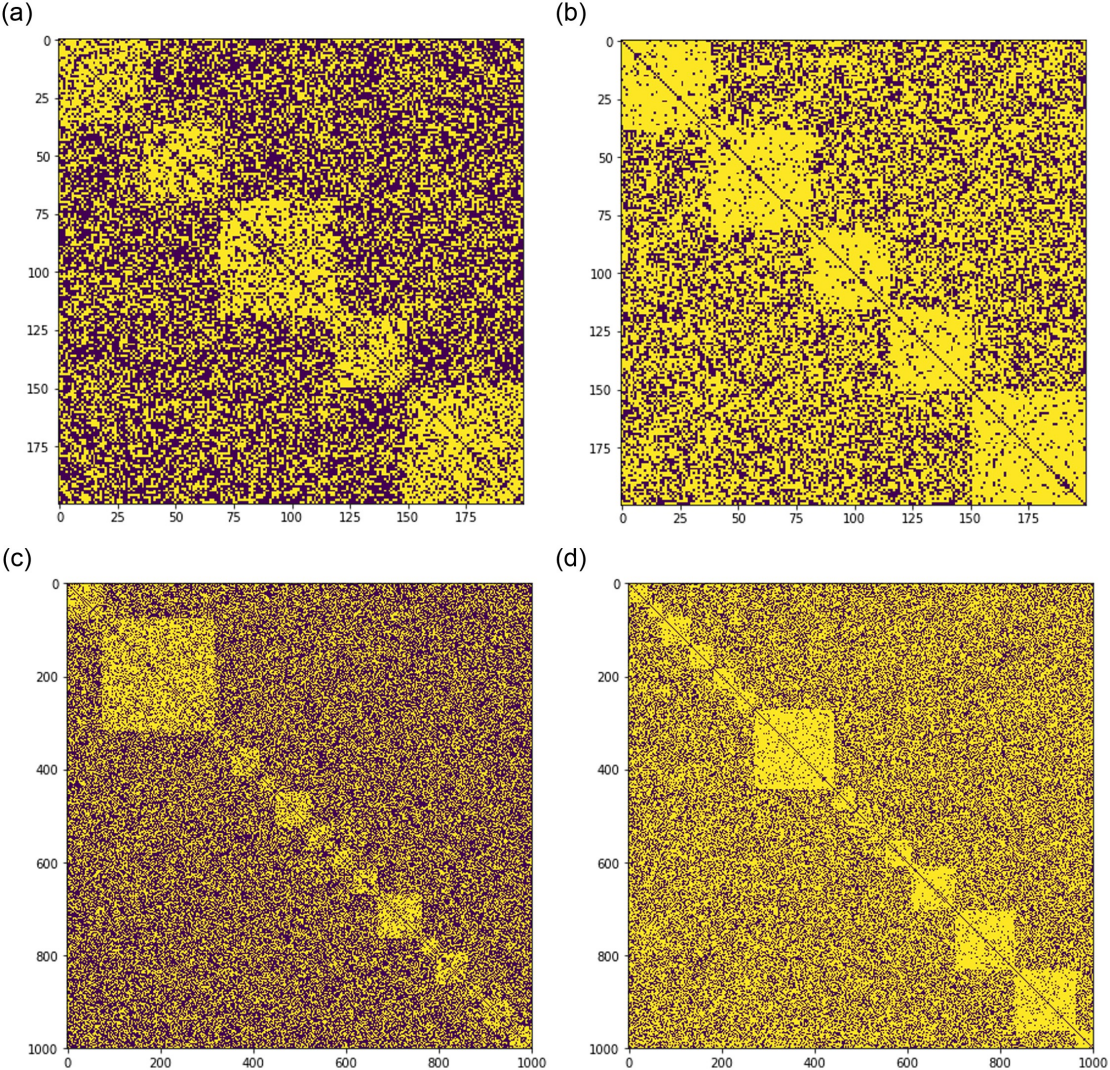
Samples of synthetically generated network matrices (white pixels represent unities, and dark ones, zeros). The number of nodes is *N* and the number of communities is *K*. Values *p*, *q* are the probabilities of drawing edges within-community and between communities, respectively. Specifically, *p* = 0.7, *q* = 0.4, *N* = 200, *K* = 5 at (a), *p* = 0.9, *q* = 0.6, *N* = 200, *K* = 5 at (b), *p* = 0.7, *q* = 0.4, *N* = 1000, *K* = 15 at (c), and *p* = 0.9, *q* = 0.6, *N* = 1000, *K* = 15 at (d).

Every within-community edge is drawn with the probability *p*, independently of other edges. Similarly, any between- community edge is drawn independently with the probability *q*. Fig 1 illustrates similarity matrices for generated networks at *p* = 0.7, 0.9 and *q* = 0.4, 0.6. The upper pane in the Figure visualizes a network with 200 nodes and five communities, whereas the lower pane presents 15 communities at 1000 nodes.

**Fig 2:**
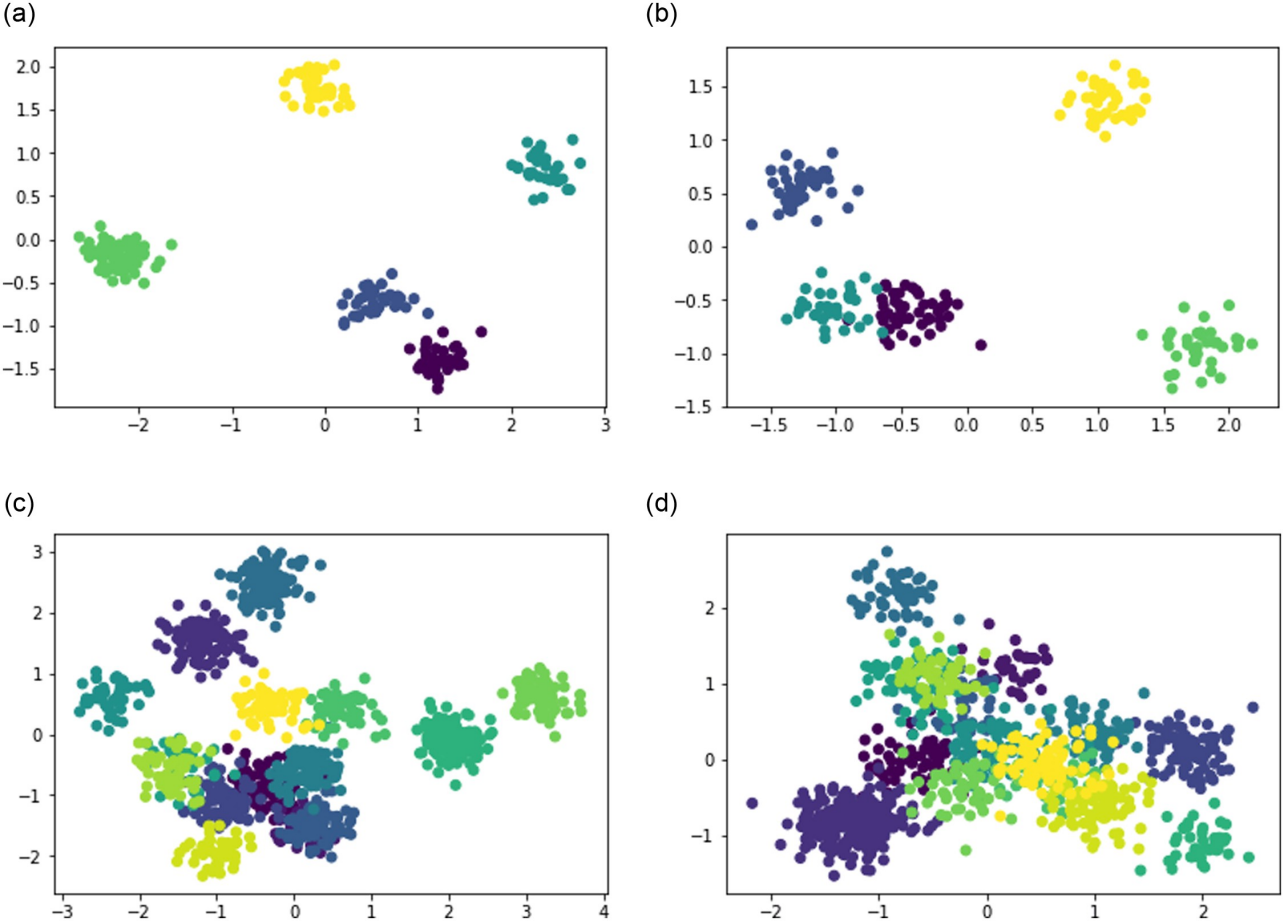
Samples of synthetically generated clusters at quantitative features; *N* is the number of nodes, *K* is the number of communities, and *α* is the parameter of cluster intermix. The parameter values are: *α* = 0.9, *N* = 200, *K* = 5 at (a); *α* = 0.7, *N* = 200, *K* = 5 at (b); *α* = 0.9, *N* = 1000, *K* = 15 at (c); and *α* = 0.7, *N* = 1000, *K* = 15 at (d).

*3.2.2.2 Generating quantitative features*. To model quantitative features, we use conventional Gaussian distributions as within-cluster density functions. We apply design proposed in [45]. Each cluster is generated from a Gaussian distribution whose covariance matrix is diagonal with diagonal values uniformly random in the range [0.05, 0.1]—they specify the cluster’s spread. Each component of the cluster center is generated uniformly random from the range *α*[−1, +1], where *α* ∈ *A* controls the cluster intermix. Indeed, the smaller the *α*, the greater the chance that points from a cluster fall within the spreads of other clusters. Fig 2 illustrates examples of the generated data sets for *α* = 0.7 and *α* = 0.9. The upper pane in the Figure visualizes a feature-rich network with 200 nodes and five communities, whereas the lower pane presents a synthetic dataset with 15 communities at 1000 nodes.

In addition to cluster intermix, the possibility of presence of noise in data also is taken into account. Uniformly random noise features from an interval defined by the maximum and minimum values are generated. In this way, 50% of the original data with noise features are replicated.

*3.2.2.3 Generating categorical features*. To model categorical features, the number of subcategories for each category is randomly chosen from the set {2, 3, …, *L*} where *L* = 10 for small-size networks and *L* = 15 for medium-size networks. Then, given the number of communities, *K*, and the numbers of entities, *N*_*k*_ for (*k* = 1, …, *K*); the cluster centers are generated randomly so that no two centers may coincide at more than 50% of features.

Once a center of *k*-th cluster, *c*_*k*_ = (*c*_*kv*_), is specified, *N*_*k*_ entities of this cluster are generated as follows. Given a pre-specified threshold of intermix, *ϵ* between 0 and 1, for every pair (*i*, *v*), *i* = 1: *N*_*k*_; *v* = 1: *V*, a uniformly random real number *r* between 0 and 1 is generated. If *r* > *ϵ*, the entry *x*_*iv*_ is set to be equal to *c*_*kv*_; otherwise, *x*_*iv*_ is taken randomly from the set of subcategories specified for feature *v*.

Consequently, all entities in cluster *k*-th coincide with its center, up to rare errors if *ϵ* is large enough. The smaller the epsilon, the more diverse, and thus intermixed, would be the generated entities.

To generate a feature-rich network combining categorical and quantitative features, we divide the number of features in two approximately equal parts, one to consist of quantitative features, the other, of categorical features. Each part is filled in independently, according to the schemes described above.

### 3.3 Data pre-processing

The results of ICESi and SEFNAC methods depend on how the data are standardized. Unfortunately, no theoretical foundations have been developed so far for the issues of data standardization. We describe here two popular methods of standardization for feature data and two standardization methods for network data.

Noteworthy to add that to convert feature data to similarity data, preprocessing them with a standardization is a must. And this is the reason to consider feature standardization methods here.

For features, the two following standardization methods are taken into consideration:

1. Z-scoring: each of the features is centered by subtraction of its mean from all its values, and then normalized by dividing over its standard deviation.
2. Range standardization: each of the features is centered by subtraction of its mean from all its values, and then normalized by dividing over its range, that is, the difference between its maximum and minimum.

For the networks, consider the two following normalization methods:

1. Modularity: Given an *N* × *N* similarity matrix *P* = (*p*_*ij*_), compute summary values $p_{i+}=\sum_{j=1}^{N}p_{ij}$, $p_{+j}=\sum_{i=1}^{N}p_{ij}$, $p_{++}=\sum_{i,j=1}^{N}p_{ij}$ and random interaction scores *t*_*ij*_ = *p*_*i*+_ *p*_+*j*_/*p*_++_. Clean link scores from random interactions by changing *p*_*ij*_ for *p*_*ij*_ − *t*_*ij*_.
2. Uniform shift: Compute the mean link score $\pi =\sum_{i,j=1}^{N}p_{ij}/N^{2}$; change all *p*_*ij*_ for *p*_*ij*_ − *π*.

### 3.4 Evaluation criterion

To compare results found by clustering algorithms, we use most popular metrics of similarity between partitions: 1) The Adjusted Rand Index (ARI) [46], and 2) the Normalised Mutual Information (NMI) [47, 48]. However, in this paper, we report only the ARI values for the sake of convenience because these two measures lead to similar conclusions. Therefore, we define here ARI only.

Let us recall the concept of contingency table from statistics. Given two partitions of the node set *I*, *S* = {*S*_1_, *S*_2_, …, *S*_*K*_} and *T* = {*T*1, *T*_2_, …, *T*_*L*_}, the contingency table is a two-way table, whose rows correspond to parts *S*_*k*_ (*k* = 1, 2, …, *K*) of *S*, and columns, to parts *l* = 1, 2, …, *L* of *T*, so that its (*k*, *l*)-th entry is *n*_*kl*_ = |*S*_*k*_ ∩ *T*_*l*_|, the frequency of (*k*, *l*) co-occurrences. The so-called marginal row and marginal column are defined as $a_{k}=\sum_{l=1}^{L}n_{kl}=\left|S_{k}\right|$ and $b_{l}=\sum_{k=1}^{K}n_{kl}=\left|T_{l}\right|$.

The Adjusted Rand Index is defined as:

ARI(S,T)=∑k,l(nkl2)-[∑k(ak2)∑l(bl2)]/(N2)12[∑k(ak2)+∑l(bl2)]-[∑k(ak2)∑l(bl2)]/(N2)] (31)

The closer the value of ARI to unity, the better the match between the two partitions; ARI = 1.0 shows that *S* = *T*. If one of the partitions consists of just one part, the set *I* itself, then ARI = 0. Cases at which ARI is negative may occur too; but these authors have observed them only at specially defined, ‘dual’, pairs of partitions (see in [45]).

To make ARI values more operational, we consider a model confusion example from [4], p. 246. This example operates with two partitions, {*S*_1_, *S*_2_} and {*T*_1_, *T*_2_} dividing *I* in two equal-sized parts each, so that the contingency table of the co-occurrence relative frequencies looks like Table 6.

**Table 6:** Model confusion data for ARI.

Parts	*T*_1_	*T*_2_	Total
*S*_1_	1/2 − *δ*	*δ*	1/2
*S*_2_	*δ*	1/2 − *δ*	1/2
Total	1/2	1/2	1

Here the *δ* value expresses the share of errors at predicting part *T*_*k*_ from part *S*_*k*_, *k* = 1, 2, so that the total error rate is 2*δ*.

To analytically express ARI in this example, we need to reformulate the ARI formula (31) in terms of ordered pairs. The ARI in (31) is based on accounting the numbers of pairs of elements either belonging to the same part in a partition or not. For any subset of *m* elements, the binomial values (m2) in (31) count the number of unordered pairs in the subset, whereas the number of ordered pairs is equal to *m*^2^. Therefore, we are going to change the binomials in (31), (m2) for their equivalent counterparts *m*^2^. Let *p*_*kl*_, *s*_*k*_, *t*_*l*_ denote the proportions of *I* entities in *S*_*k*_ ∩ *T*_*l*_, *S*_*k*_, *T*_*l*_, respectively (*k*, *l* = 1, 2). Then

$$\begin{array}{c} ARI\left(S,T\right)=\frac{C-A*B}{(A+B)/2-A*B} \end{array}$$ (32)

where $A=\sum_{k}s_{k}^{2}$, $B=\sum_{l}t_{l}^{2}$, $C=\sum_{k,l}p_{kl}^{2}$.

In our example, obviously *A* = *B* = 1/2. The *C* value is equal to *C* = 2 * [*δ*^2^ + (1/2 − *δ*)^2^] = 1/2 − 2*δ* + 4*δ*^2^. Then the numerator in (32) is equal to *C* − *A* * *B* = 1/4 − 2*δ* + 4*δ*^2^ = (1/2 − 2*δ*)^2^. Taking into account that the denominator is equal to 1/4, we arrive at equation *ARI*(*S*, *T*) = (1 − 4*δ*)^2^. This allows us to calibrate ARI values using error rates in our model confusion example (see Table 7).

**Table 7:** A calibration table relating the error rate in the model confusion example with the corresponding ARI values.

*δ*, per cent	Error rate per cent	ARI value
1	2	0.92
2.5	5	0.81
5	10	0.64
7.5	15	0.49
10	20	0.36
15	30	0.16
20	40	0.04
25	50	0

It should be mentioned that there exist different approaches to evaluation of results of community detection methods, involving both internal aspects (such as the proportion of immediate neighbours of a community member belonging to the same community) and external aspects (such as similarity between community size distributions). An interested reader is referred to papers [49–51] at which they can find necessary details.

## 4 Experimental comparison of the methods under consideration

### 4.1 Comparison of methods over real-world datasets

In this section we compare the performance ICESi methods with that of SEFNAC, SIAN and CESNA at the five real-world datasets described above in subsection 3.2. All the algorithms are run starting from random configurations ten times at each of the datasets.

Those pre-processing methods that lead, on average, to the larger ARI values, have been chosen for the least-squares methods, as presented in Table 8.

**Table 8:** Standardization options chosen for the least-squares community extraction methods at the real world datasets.

Dataset	SEFNAC		ICESiss		ICESisn		ICESins		ICESinn	
	Y	P	R	P	R	P	R	P	R	P
Malaria HVR6	Zs	Us	Zs	Us	Zs	Mo	Rs	Mo	Zs	Us
Lawyers	Rs	Us	Rs	Us	Zs	Mo	Rs	Mo	Rs	Us
World Trade	Rs	Rs	Rs	Rs	Rs	Us	Zs	Zs	Zs	Zs
Parliament	Zs	Mo	Zs	Mo	Rs	Us	Rs	Us	Zs	Mo
COSN	Zs		Zs	Us	Zs	Mo	Zs	Us	Rs	Us

Symbols Zs, Us, Mo, and Us stand for the Z-scoring, Range standardization, Modularity and Uniform shift, respectively.

The Table 9 presents the results of comparison of all the algorithms under consideration over real-world datasets.

**Table 9:** The comparison of CESNA, SIAN, SEFNAC and ICESi methods at Real-world data sets; average values of ARI are presented over 10 random initialization.

Dataset	CESNA	SIAN	SEFNAC	ICESiss	ICESins	ICESinn	ICESisn
HRV6	0.20(0.00)	0.39(0.29)	0.49(0.11)	**0.62(0.00)**	**0.62(0.00)**	0.59(0.01)	**0.62(0.00)**
Lawyers	0.28(0.00)	0.59(0.04)	**0.60(0.09)**	0.420(0.07)	0.51(0.01)	0.51(0.01)	0.35(0.11)
World Trade	0.13(0.00)	0.10(0.01))	0.29(0.09)	**0.47(0.13)**	0.37(0.02)	0.36(0.02)	**0.47(0.15)**
Parliament	0.25(0.00)	**0.79(0.12)**	0.28(0.01)	0.00(0.00)	0.00(0.00)	0.34(0.03)	0.00(0.00)
COSN	0.44(0.00)	0.75(0.00)	0.72(0.02)	0.63(0.13)	**0.83(0.00)**	0.50(0.01)	0.76(0.03)

The best results are highlighted in bold-face and second ones are under-lined.

As one can see, SIAN is the winner for Parliament dataset; also it takes the second place for COSN dataset. SEFNAC wins the competition on Lawyer dataset. The proposed methods ICESiss, ICESins, and ICESisn, win the competition on HRV6 dataset. Moreover, two of these, ICESiss and ICESisn, also win the competition on World Trade dataset. The ICESins wins the competition on COSN dataset.

These results show that converting feature data to similarity data, over real-world data sets, may lead to more accurate cluster recovery results indeed. However, we cannot say at this stage, what characteristics of the datasets may lead to a successful application of this or that method.

### 4.2 Comparison of methods over synthetic datasets with categorical features

Tables 10 and 11 report of the experimental results of comparison of all of the algorithms under consideration over networks with categorical features at small-sizes and medium sizes, respectively.

**Table 10:** The comparison of CESNA, SIAN, SEFNAC and ICESi methods at small-size synthetic data sets with categorical attributes: The average ARI index and its standard deviation over 10 different data sets.

*p*, *q*, *ϵ*	CESNA	SIAN	SEFNAC	ICESiss	ICESins	ICESinn	ICESisn
0.9, 0.3, 0.9	**1.00(0.00)**	0.554(0.285)	0.994(0.008)	0.932(0.035)	0.886(0.069)	0.833(0.113)	0.931(0.032)
0.9, 0.3, 0.7	0.948(0.105)	0.479(0.289)	**0.974(0.024)**	0.623(0.104)	0.529(0.135)	0.504(0.144)	0.581(0.102)
0.9, 0.6, 0.9	0.934(0.075)	0.320(0.255)	**0.965(0.013)**	0.903(0.056)	0.887(0.050)	0.836(0.095)	0.918(0.029)
0.9, 0.6, 0.7	**0.902(0.063)**	0.110(0.138)	0.750(0.117)	0.582(0.104)	0.521(0.067)	0.478(0.081)	0.554(0.103)
0.7, 0.3, 0.9	0.965(0.078)	0.553(0.157)	**0.975(0.018)**	0.917(0.047)	0.858(0.141)	0.835(0.098)	0.905(0.046)
0.7, 0.3, 0.7	**0.890(0.138)**	0.508(0.211)	0.870(0.067)	0.520(0.113)	0.493(0.095)	0.452(0.097)	0.517(0.098)
0.7, 0.6, 0.9	0.506(0.101)	0.047(0.087)	0.896(0.067)	**0.917(0.030)**	0.863(0.063)	0.803(0.102)	0.890(0.065)
0.7, 0.6, 0.7	0.202(0.081)	0.030(0.040)	**0.605(0.091)**	0.591(0.097)	0.589(0.118)	0.507(0.170)	0.597(0.113)

The best results are highlighted in bold-face and second ones are under-lined.

**Table 11:** The comparison of CESNA, SIAN, SEFNAC and ICESi methods at medium-size synthetic data sets with categorical attributes: The average ARI index and its standard deviation over 10 different data sets.

*p*, *q*, *α*	CESNA	SIAN	SEFNAC	ICESiss	ICESins	ICESinn	ICESisn
0.9, 0.3, 0.9	0.894(0.053)	0.000(0.000)	**1.000(0.000)**	0.996(0.005)	0.965(0.038)	0.976(0.035)	0.995(0.005)
0.9, 0.3, 0.7	0.849(0.076)	0.000(0.000)	**0.996(0.005)**	0.895(0.045)	0.800(0.045)	0.772(0.059)	0.904(0.033)
0.9, 0.6, 0.9	0.632(0.058)	0.000(0.000)	**0.998(0.002)**	**0.998(0.002)**	0.976(0.022)	0.981(0.018)	**0.998(0.002)**
0.9, 0.6, 0.7	0.474(0.089)	0.000(0.000)	**0.959(0.032)**	0.858(0.060)	0.800(0.045)	0.798(0.051)	0.874(0.047)
0.7, 0.3, 0.9	0.764(0.068)	0.026(0.077)	**1.000(0.001)**	0.990(0.013)	0.981(0.020)	0.975(0.028)	0.990(0.013)
0.7, 0.3, 0.7	0.715(0.128)	0.000(0.000)	**0.993(0.002)**	0.903(0.023)	0.822(0.070)	0.800(0.077)	0.901(0.029)
0.7, 0.6, 0.9	0.060(0.024)	0.000(0.000)	0.998(0.001)	**0.999(0.001)**	0.978(0.027)	0.971(0.028)	**0.999(0.001)**
0.7, 0.6, 0.7	0.016(0.008)	0.000(0.000)	**0.909(0.035)**	0.875(0.039)	0.727(0.072)	0.753(0.088)	0.861(0.047)

The best results are highlighted in bold-face and second ones are under-lined.

By comparing the two tables one can see how the performances of model-based CESNA and SIAN algorithms deteriorate when one moves from the small-size to the medium-size. CESNA is a leader, on par with SEFNAC, at small-size datasets, to move out of the winning places, with a few real poor results at less tight structures, at medium-size datasets. SIAN moves from a mediocre performance at small-sizes to really poor results at the medium-sizes. This might have happened because of the assumption made about the sparsity of networks. The convergence issues can be another reason of the poor performance.

The SEFNAC algorithm is a leader at the small-size data and is the undisputed leader at the medium-size data. It is quite impressive, how well the SEFNAC faces up the challenges of loose structures at larger values of *q* and smaller values of *α*. Nevertheless, ICESiss and ICESisn rise to the challenge at medium-sized data, at which they manage to win in two out of eight settings. However, they are less steady at the worst combination, *q* = 0.6 and *α* = 0.7.

### 4.3 Complexity issues for ICESi methods

ICESi methods are computationally intensive: they compute and compare values of the criterion *G* while finding a node to be added to a current community. In the current implementation, the part of criterion *G* for the nonsummable mode is computed in a vectorized form, whereas the part corresponding to the summable mode requires a nested “for” loop, which takes a longer time, by the order of *N*. Then the total time for execution of CESinn is proportional to *N*^2^ and it is proportional to *N*^3^, for execution of CESiss. Indeed, to find a community, CESi adds a number of nodes proportional to *N*, and selecting a node to be added at a step, requires a number of tries proportional to *N* too. We do not take into account the number of communities found, because it is limited by a constant.

To check whether the execution times go in line with those by the other algorithms under consideration, we took a common ground, synthetic networks with categorical features only, and ran all the algorithms at both small-sized synthetic datasets and medium-sized synthetic datasets. The computing time should not much depend on the parameter setting; thus, we selected two out of our standard eight settings: (a) (*p*, *q*, *ϵ*) = (0.9, 0.3, 0.9) at which the community structure is maximally sharp; and (b) (*p*, *q*, *ϵ*) = (0.7, 0.6, 0.7) at which the community structure is maximally blurred.

In practice, the computation time depends on the computing system, so that only relative comparisons can be meaningful. The times reported in the following Table 12 have been observed at a desktop computer Intel(R) (Core(TM) i9-9900K CPU /@ 3.60GHz, RAM: 64 GB, HD: 1TB SSD) under Ubuntu 18.0 Operation System.

**Table 12:** The execution time of methods under consideration at synthetic networks with categorical features at the nodes. The average of 10 different data sets of the same setting is reported in seconds.

	CESNA		SIAN		SEFNAC		ICESiss		ICESins		ICESinn		ICESisn	
*p*, *q*, *ϵ*	small	medium	small	medium	small	medium	small	medium	small	medium	small	medium	small	medium
0.9, 0.3, 0.9	0.442	38.265	95.949	856.785	4.647	492.006	8.224	1038.619	3.390	326.141	1.816	78.142	3.272	339.563
0.7, 0.6, 0.7	0.699	83.961	335.198	2674.541	3.652	476.251	7.822	1186.691	3.306	580.418	1.827	92.482	3.097	407.207

The table shows that CESNA is the fastest method and SIAN the slowest method out of the methods under consideration: their timings differ by two orders of magnitude. All the ICESi methods fall within the boundaries set by CESNA and SIAN. Rather expectedly, ICESInn is the fastest and ICESIss the slowest among them. Note that ICESinn approaches the speed of CESNA, whereas the speed of ICESiss is closer to that of SIAN.

## 5 Experimental validation of ICESi methods

### 5.1 Experimental results for ICESi methods at various feature scales

#### 5.1.1 Chosen data standardization options

Considering data standardization options defined in Section 3.3, we chose those leading to best data recovery results at the corresponding data formats. Based on a thorough computational experiment, our choices can be described as follows: the combination of Range standardization and Uniform methods is the combination of data pre-processing techniques for all the ICESi methods (with ss or sn or ns or nn ending) at almost all synthetic data generated, except for the following cases. The combination of Z-scoring and Modularity should be applied for ICESiss at networks with quantitative or mixed-scale features, as well as for ICESinn at networks with categorical or mixed-scale features. The combination of Z-scoring and Uniform methods should be applied for ICESins at networks with categorical features.

#### 5.1.2 ICESi at synthetic datasets with quantitative features

Table 13 shows the performance of ICESi methods by applying the selected pre-processing techniques at small-size networks with quantitative features.

**Table 13:** The performance of ICESi methods at small-size networks with quantitative features at the nodes; with selected data pre-processing for each algorithm: The average ARI index and its standard deviation over 10 different data sets.

	ICESiss		ICESins		ICESinn		ICESisn	
Setting *p*, *q*, *α*	ARI mean(std)	K mean(std)	ARI mean(std)	K mean(std)	ARI mean(std)	K mean(std)	ARI mean(std)	K mean(std)
0.9, 0.3, 0.9	0.869(0.140)	4.900(1.044)	0.994(0.014)	5.100(0.300)	0.953(0.073)	4.700(0.458)	**1.000(0.000)**	**5.000(0.000)**
0.9, 0.3, 0.7	0.774(0.138)	4.600(1.114)	0.998(0.004)	5.000(0.000)	0.950(0.109)	4.700(0.640)	**1.000(0.000)**	**5.000(0.000)**
0.9, 0.6, 0.9	0.732(0.177)	4.500(1.565)	0.765(0.233)	3.800(1.166)	0.729(0.208)	3.600(1.020)	**0.858(0.181)**	**4.300(0.900)**
0.9, 0.6, 0.7	0.783(0.153)	5.100(1.221)	**0.873(0.137)**	**4.400(0.663)**	0.822(0.122)	4.300(0.640)	0.840(0.100)	4.800(0.600)
0.7, 0.3, 0.9	0.698(0.093)	4.100(0.943)	**0.939(0.074)**	**4.700(0.458)**	0.870(0.124)	4.300(0.640)	0.928(0.074)	4.600(0.490)
0.7, 0.3, 0.7	0.833(0.133)	5.500(1.285)	0.954(0.075)	4.800(0.400)	**0.960(0.075)**	**4.800(0.400)**	0.947(0.073)	4.700(0.458)
0.7, 0.6, 0.9	**0.771(0.144)**	**4.800(1.077)**	0.694(0.134)	4.700(2.571)	0.696(0.126)	5.100(2.625)	0.146(0.176)	10.100(2.468)
0.7, 0.6, 0.7	**0.707(0.127)**	**5.200(0.600)**	0.659(0.188)	4.700(1.269)	0.647(0.163)	6.000(1.265)	0.060(0.096)	11.100(1.640)

The best results are highlighted in bold-face.

Table 14 represents the results on small-size networks with quantitative features at the nodes with 50% noise features.

**Table 14:** The performance of ICESi methods at small-size networks with quantitative features at the nodes with 50% noise features replicated; with selected data pre-processing for each algorithm: The average ARI index and its standard deviation over 10 different data sets.

	ICESiss		ICESins		ICESinn		ICESisn	
Setting *p*, *q*, *α*	ARI mean(std)	K mean(std)	ARI mean(std)	K mean(std)	ARI mean(std)	K mean(std)	ARI mean(std)	K mean(std)
0.9, 0.3, 0.9	0.688(0.157)	6.500(1.910)	0.731(0.147)	5.000(1.265)	0.948(0.081)	4.700(0.458)	**1.000(0.000)**	**5.000(0.000)**
0.9, 0.3, 0.7	0.531(0.115)	6.200(0.872)	0.639(0.180)	4.800(1.536)	0.962(0.078)	4.800(0.400)	**0.999(0.003)**	**5.000(0.000)**
0.9, 0.6, 0.9	0.700(0.112)	6.200(1.077)	0.667(0.175)	4.700(1.616)	0.701(0.217)	3.700(1.100)	**0.796(0.199)**	**4.100(0.943)**
0.9, 0.6, 0.7	0.566(0.105)	7.000(1.789)	0.700(0.105)	5.300(0.781)	**0.824(0.140)**	**4.300(0.900)**	0.804(0.145)	4.400(1.020)
0.7, 0.3, 0.9	0.738(0.107)	5.500(1.285)	0.682(0.114)	4.800(1.400)	0.841(0.143)	4.200(0.748)	**0.944(0.066)**	**4.700(0.458)**
0.7, 0.3, 0.7	0.517(0.133)	6.200(1.536)	0.671(0.110)	5.000(1.183)	0.934(0.099)	4.700(0.458)	**0.946(0.080)**	**4.700(0.458)**
0.7, 0.6, 0.9	**0.678(0.120)**	**5.600(1.114)**	0.628(0.168)	4.300(1.792)	0.654(0.107)	5.300(2.238)	0.196(0.186)	9.700(2.759)
0.7, 0.6, 0.7	0.566(0.108)	6.900(0.943)	**0.645(0.190)**	**4.800(0.872)**	0.622(0.148)	6.400(1.356)	0.129(0.133)	10.400(1.960)

The best results are highlighted in bold-face.

The Tables 13 and 14 show rather similar patterns, although indeed the ARI values in the latter table are slightly smaller than in the former: the noise inserted has not destroyed the structures recovered. Moreover, the noise made the supremacy of ICESisn somewhat more pronounced, as the corresponding columns in the tables clearly demonstrate. However, the ICESisn fails at the difficult network link setting with *p* = 0.7 and *q* = 0.6 (see the two last lines in the tables). This can be attributed to the fact that the method finds by far more clusters than there have been generated, about 10, with a large variance. The other three methods obtain much less clusters at this setting, bringing them to decent recovery results. Overall, one may find method ICESinn bringing rather robust recovery results. One should not miss the fact that both the methods mentioned are based on the nonsummable link usage.

Tables 15 and 16 present results obtained with the four methods over the medium-size networks with quantitative features; the latter table refers to the case of 50% noise features inserted.

**Table 15:** The performance of ICESi methods at medium-size networks with quantitative features at the nodes; with selected data pre-processing for each algorithm: The average ARI index and its standard deviation over 10 different data sets.

	ICESiss		ICESins		ICESinn		ICESisn	
Setting *p*, *q*, *α*	ARI mean(std)	K mean(std)	ARI mean(std)	K mean(std)	ARI mean(std)	K mean(std)	ARI mean(std)	K mean(std)
0.9, 0.3, 0.9	0.666(0.102)	8.700(1.005)	**0.968(0.046)**	**14.200(0.980)**	0.747(0.102)	10.300(0.900)	0.931(0.071)	13.400(1.625)
0.9, 0.3, 0.7	0.604(0.086)	8.800(1.600)	**0.907(0.105)**	**13.300(1.616)**	0.718(0.131)	10.000(2.098)	0.868(0.164)	12.200(3.092)
0.9, 0.6, 0.9	**0.623(0.120)**	**8.800(1.536)**	0.587(0.149)	9.300(1.552)	0.559(0.132)	8.500(1.803)	**0.623(0.075)**	**9.700(1.269)**
0.9, 0.6, 0.7	0.531(0.113)	8.800(1.720)	0.609(0.188)	9.300(1.676)	0.520(0.155)	8.400(1.428)	**0.627(0.080)**	**10.400(2.010)**
0.7, 0.3, 0.9	0.610(0.105)	8.700(1.100)	0.715(0.133)	10.100(1.221)	0.589(0.168)	9.100(1.446)	**0.765(0.123)**	**10.300(1.552)**
0.7, 0.3, 0.7	0.493(0.190)	57.800(146.743)	0.702(0.178)	10.800(1.990)	0.577(0.135)	8.800(1.536)	**0.750(0.121)**	**10.800(2.040)**
0.7, 0.6, 0.9	**0.625(0.136)**	**8.900(1.300)**	0.544(0.089)	7.900(0.943)	0.523(0.083)	7.900(0.943)	0.195(0.204)	13.100(3.300)
0.7, 0.6, 0.7	**0.602(0.103)**	**9.200(1.536)**	0.475(0.096)	8.300(1.005)	0.457(0.095)	9.000(1.612)	0.040(0.111)	15.700(2.759)

The best results are highlighted in bold-face.

**Table 16:** The performance of ICESi methods at medium-size networks with quantitative features at the nodes with 50% noise features replicated; with selected data pre-processing for each algorithm: The average ARI index and its standard deviation over 10 different data sets.

	ICESiss		ICESins		ICESinn		ICESisn	
Setting *p*, *q*, *α*	ARI mean(std)	K mean(std)	ARI mean(std)	K mean(std)	ARI mean(std)	K mean(std)	ARI mean(std)	K mean(std)
0.9, 0.3, 0.9	0.267(0.210)	17.100(3.300)	0.474(0.093)	7.800(0.980)	0.693(0.107)	9.900(1.446)	**0.948(0.052)**	**13.700(1.005)**
0.9, 0.3, 0.7	0.369(0.239)	17.200(4.556)	0.503(0.081)	8.900(1.136)	0.703(0.134)	10.400(1.908)	**0.829(0.199)**	**11.900(3.270)**
0.9, 0.6, 0.9	0.118(0.134)	14.900(4.011)	0.447(0.120)	8.000(2.098)	0.427(0.097)	9.300(1.418)	**0.549(0.129)**	**9.100(2.119)**
0.9, 0.6, 0.7	0.048(0.092)	17.500(1.857)	0.427(0.075)	9.000(1.414)	0.292(0.095)	10.100(1.446)	**0.615(0.127)**	**10.500(1.432)**
0.7, 0.3, 0.9	0.447(0.170)	13.200(3.429)	0.440(0.122)	8.200(1.720)	0.480(0.147)	9.000(1.342)	**0.722(0.130)**	**9.800(1.778)**
0.7, 0.3, 0.7	0.452(0.163)	12.800(3.124)	0.462(0.115)	8.100(1.700)	0.459(0.094)	9.400(1.200)	**0.751(0.116)**	**10.700(1.792)**
0.7, 0.6, 0.9	0.272(0.127)	12.600(1.960)	**0.571(0.122)**	**8.200(0.872)**	0.363(0.111)	9.600(1.281)	0.210(0.184)	12.900(2.809)
0.7, 0.6, 0.7	0.135(0.135)	13.300(2.532)	**0.483(0.094)**	**8.900(1.446)**	0.245(0.136)	10.400(0.663)	0.086(0.093)	14.700(2.283)

The best results are highlighted in bold-face.

These tables do show both similarities with and differences from those at small-size datasets. The ICESisn is superior again, with the superiority getting more pronounced in the latter table, at the presence of noise features, although the respective ARI values are somewhat smaller here. Once again, ICESins fails at the two last lines corresponding to the lousy structure at *p* = 0.7, *q* = 0.6. This time, however, only one of the three other methods holds on: ICESiss, at the data with no noise, and ICESins at noise present. It ought to be mentioned that these are the only cases at which the presence of noise is not that destructive.

#### 5.1.3 ICESi at synthetic datasets with categorical features

Tables 17 and 18 show the performance of the four methods under consideration over networks with categorical features, the small-size and medium size, respectively.

**Table 17:** The performance of ICESi methods at small-size networks with categorical features at the nodes; with selected data pre-processing for each algorithm: The average ARI index and its standard deviation over 10 different data sets.

	ICESiss		ICESins		ICESinn		ICESisn	
Setting *p*, *q*, *α*	ARI mean(std)	K mean(std)	ARI mean(std)	K mean(std)	ARI mean(std)	K mean(std)	ARI mean(std)	K mean(std)
0.9, 0.3, 0.9	0.930(0.026)	6.100(0.700)	0.886(0.069)	6.200(0.748)	0.833(0.113)	5.900(0.831)	**0.931(0.032)**	**6.400(0.490)**
0.9, 0.3, 0.7	**0.599(0.117)**	**8.300(0.781)**	0.529(0.135)	8.100(0.539)	0.504(0.144)	8.000(1.095)	0.581(0.102)	8.100(0.700)
0.9, 0.6, 0.9	0.904(0.061)	6.400(0.800)	0.887(0.050)	6.500(1.204)	0.836(0.095)	6.000(0.775)	**0.918(0.029)**	**6.300(0.900)**
0.9, 0.6, 0.7	**0.569(0.085)**	**7.800(0.980)**	0.521(0.067)	8.200(1.536)	0.478(0.081)	7.700(0.640)	0.554(0.103)	8.300(1.100)
0.7, 0.3, 0.9	**0.925(0.029)**	**6.500(0.500)**	0.858(0.141)	6.400(0.490)	0.835(0.098)	6.200(0.600)	0.905(0.046)	6.200(0.400)
0.7, 0.3, 0.7	**0.520(0.115)**	**8.000(1.095)**	0.493(0.095)	8.000(0.632)	0.452(0.097)	7.400(0.800)	0.517(0.098)	8.200(0.980)
0.7, 0.6, 0.9	**0.921(0.031)**	**6.600(0.663)**	0.863(0.063)	6.300(0.640)	0.803(0.102)	6.100(0.943)	0.890(0.065)	6.600(0.663)
0.7, 0.6, 0.7	**0.605(0.109)**	**8.600(1.020)**	0.589(0.118)	8.500(0.806)	0.507(0.170)	7.700(1.005)	0.597(0.113)	8.300(0.640)

The best results are highlighted in bold-face.

**Table 18:** The performance of ICESi methods at medium-size networks with categorical features at the nodes; with selected data pre-processing for each algorithm: The average ARI index and its standard deviation over 10 different data sets.

	ICESiss		ICESins		ICESinn		ICESisn	
Setting *p*, *q*, *α*	ARI mean(std)	K mean(std)	ARI mean(std)	K mean(std)	ARI mean(std)	K mean(std)	ARI mean(std)	K mean(std)
0.9, 0.3, 0.9	**0.996(0.005)**	**14.900(0.300)**	0.965(0.038)	14.400(0.663)	0.976(0.035)	16.500(1.204)	0.995(0.005)	14.900(0.300)
0.9, 0.3, 0.7	0.895(0.045)	18.600(1.685)	0.800(0.045)	17.200(1.249)	0.772(0.059)	14.200(0.980)	**0.904(0.033)**	**18.600(2.200)**
0.9, 0.6, 0.9	**0.998(0.002)**	**15.000(0.000)**	0.976(0.022)	14.300(0.781)	0.981(0.018)	16.700(1.345)	**0.998(0.002)**	**15.000(0.000)**
0.9, 0.6, 0.7	0.858(0.060)	17.800(1.077)	0.800(0.045)	17.200(1.249)	0.798(0.051)	14.600(0.490)	**0.874(0.047)**	17.700(1.100)
0.7, 0.3, 0.9	**0.990(0.013)**	**14.800(0.400)**	0.981(0.020)	14.600(0.490)	0.975(0.028)	14.500(0.806)	**0.990(0.013)**	**14.800(0.400)**
0.7, 0.3, 0.7	**0.903(0.023)**	**18.600(1.497)**	0.822(0.070)	17.300(1.269)	0.800(0.077)	17.000(1.789)	0.901(0.029)	18.800(1.327)
0.7, 0.6, 0.9	**0.999(0.001)**	**15.000(0.000)**	0.978(0.027)	14.500(0.922)	0.971(0.028)	14.200(0.980)	**0.999(0.001)**	**15.000(0.000)**
0.7, 0.6, 0.7	**0.875(0.039)**	**18.400(0.663)**	0.727(0.072)	16.300(1.100)	0.753(0.088)	16.900(1.868)	0.861(0.047)	18.800(1.400)

The best results are highlighted in bold-face.

At these tables, ICESiss is the obvious winner. Its superiority gets overwhelming at the medium-size datasets. Indeed, one can clearly distinguish the influence of a smaller threshold *ϵ* = 0.7 at the small-size datasets. The performance of ICESiss falls down to ARI = 0.5 at this *ϵ* value. Of course the other three methods are not immune to the effect either: ARI falls even to smaller values in these cases. However, at medium-sized networks this effect does not hold any more, so that the ARI values in most cases approach a unity here for ICESiss. Perhaps such an improvement happens due to the increase in the number of samples making the clusters more homogeneous. A close follower is ICESisn, that takes over the leadership in many cases at the medium-sized data. It should be noticed that the other two methods show similar, rather high, ARI patterns in the Table 18 related to the medium-size data.

#### 5.1.4 ICESi at synthetic datasets combining quantitative and categorical features

Table 19 presents results of comparison of the four methods on small-size networks combining quantitative and categorical features at the nodes.

**Table 19:** The performance of ICESi methods at small-size networks combining quantitative and categorical features at the nodes: The average ARI index and its standard deviation over 10 different data sets.

	ICESiss		ICESins		ICESinn		ICESisn	
Setting *p*, *q*, *α*|*ϵ*	ARI mean(std)	K mean(std)	ARI mean(std)	K mean(std)	ARI mean(std)	K mean(std)	ARI mean(std)	K mean(std)
0.9, 0.3, 0.9	**0.814(0.157)**	**5.800(1.249)**	0.732(0.169)	5.900(0.831)	0.774(0.126)	5.400(0.800)	0.716(0.167)	5.100(0.831)
0.9, 0.3, 0.7	0.573(0.112)	**6.300(1.676)**	0.542(0.103)	6.300(1.487)	**0.654(0.213)**	5.900(0.539)	0.529(0.098)	5.500(1.025)
0.9, 0.6, 0.9	**0.769(0.090)**	**6.000(1.483)**	0.733(0.083)	5.800(0.872)	0.656(0.121)	6.000(0.632)	0.755(0.082)	5.500(0.671)
0.9, 0.6, 0.7	**0.576(0.117)**	**6.200(1.470)**	0.573(0.087)	5.800(0.872)	0.469(0.160)	5.900(1.044)	0.551(0.094)	5.500(0.806)
0.7, 0.3, 0.9	0.768(0.108)	5.500(0.922)	0.664(0.109)	4.400(0.917)	0.717(0.192)	5.600(0.917)	**0.780(0.124)**	**4.900(0.700)**
0.7, 0.3, 0.7	**0.601(0.089)**	**5.900(0.831)**	0.583(0.087)	5.400(0.663)	0.510(0.200)	5.800(1.600)	0.537(0.056)	5.400(0.917)
0.7, 0.6, 0.9	0.713(0.134)	6.000(1.612)	0.656(0.123)	5.200(0.980)	0.568(0.123)	5.500(1.025)	**0.739(0.120)**	**5.700(1.552)**
0.7, 0.6, 0.7	**0.571(0.129)**	**6.000(1.265)**	0.547(0.121)	5.600(0.800)	0.320(0.116)	6.100(1.700)	0.550(0.078)	6.200(1.400)

The best results are highlighted in bold-face.

ICESiss dominates in this table, with occasional interventions by the methods with the nonsummable mode for network data, ICESisn and ICESinn. Method ICESns trails behind, although with rather decent ARI values. Similar results are obtained at the case at which 50% noise features are added.

Table 20 compares the performance of the proposed methods at medium-size networks combining quantitative and categorical features.

**Table 20:** The performance of ICESi methods at medium-size networks combining quantitative and categorical features at the nodes: The average ARI index and its standard deviation over 10 different data sets.

	ICESiss		ICESins		ICESinn		ICESisn	
Setting *p*, *q*, *α*|*ϵ*	ARI mean(std)	K mean(std)	ARI mean(std)	K mean(std)	ARI mean(std)	K mean(std)	ARI mean(std)	K mean(std)
0.9, 0.3, 0.9	0.564(0.066)	11.200(0.872)	0.473(0.077)	9.700(1.005)	0.492(0.052)	9.500(0.806)	**0.744(0.081)**	**12.200(2.441)**
0.9, 0.3, 0.7	0.415(0.078)	11.100(0.943)	0.370(0.107)	9.800(1.077)	0.348(0.087)	9.900(1.136)	**0.533(0.187)**	**10.700(1.676)**
0.9, 0.6, 0.9	0.476(0.115)	10.100(1.640)	0.453(0.094)	9.500(1.803)	0.457(0.093)	9.700(1.900)	**0.479(0.056)**	**11.300(1.345)**
0.9, 0.6, 0.7	**0.391(0.069)**	**10.300(1.552)**	0.361(0.064)	10.100(1.446)	0.379(0.066)	10.500(1.285)	0.261(0.056)	12.500(1.432)
0.7, 0.3, 0.9	**0.571(0.080)**	**11.700(1.676)**	0.473(0.074)	9.800(1.166)	0.483(0.089)	9.800(1.400)	0.527(0.049)	12.000(1.732)
0.7, 0.3, 0.7	**0.448(0.059)**	**11.200(1.661)**	0.364(0.053)	10.300(2.002)	0.353(0.052)	10.300(1.487)	0.338(0.049)	12.200(1.327)
0.7, 0.6, 0.9	**0.544(0.060)**	**10.900(1.972)**	0.467(0.092)	9.800(1.166)	0.445(0.083)	9.200(0.980)	0.326(0.095)	10.700(1.676)
0.7, 0.6, 0.7	**0.387(0.051)**	**11.500(2.247)**	0.334(0.067)	10.300(1.345)	0.301(0.084)	9.800(1.166)	0.113(0.066)	11.400(2.200)

The best results are highlighted in bold-face.

Although ICESiss dominates the scene, except for the first two settings, at *p* = 0.9 and *q* = 0.3, at which ICESisn is the winner, the results look real mediocre, with ARI values somewhere between 0.4 and 0.5, corresponding to 15-20% error rates in the model confusion example in Table 7. Similar results, not shown, are obtained with 50% noise features replicated.

Overall, the performance of ICESi methods at synthetic networks with mixed scale features are not that bad, yet in this setting, ICESi results are inferior to those by SEFNAC, as reported in [34].

## 6 Conclusion and future work

This paper continues the line of research started by the authors in [34]. We explore whether the doubly-greedy least-squares approach proposed in [34] can be successfully applied to feature-rich networks at which the feature-related part is converted to a similarity matrix format. Usually, similarity data are considered as measured in the same scale so that one can meaningfully compare and sum similarity values across the entire similarity matrix (summability mode). However, there can be situations in which similarity values in one column (or row) should not be compared with the values in another column (or row)—nonsummable mode. By applying this to the two similarity matrices, that feature-generated and that native, with link scores, we come to four different summability patterns denoted in the paper by ss, ns, sn, and nn, and, accordingly to four different Iterative Community Extraction from Similarity data (ICESi) algorithms.

One of the theoretical advantages of ICESi is a Pythagorean decomposition of the data scatter in the sum of the least-squares criterion and individual cluster contributions—this allows to score the contribution of various elements of found solutions to the data scatter, which can be useful for interpretation [4]. Among practical advantages is competitiveness of the doubly-greedy approach regarding its capacity for the cluster recovery against other computational procedures (see, for example, experimental results in [32–34, 52]).

Let us take a look at general properties of the least squares model under consideration:

(i) It models the observed data rather than the way they are generated;
(ii) It is a weighted sum of two summary squared differences between the data and models with regard to the two data sources, network and features;
(iii) It involves cluster structure represented by a set of non-overlapping clusters, their intensity weights, and centers in the feature space;
(iv) It involves a hard optimization problem.

Each of these items brings forth some properties, part of them to advantage, part to disadvantage.

Let us comment, first of all, on item (i). An advantage of our model is that one can straightforwardly go for what matters most, node memberships in communities, along with quantitative evaluations of within-community link intensities and central points in the feature space. The formulation naturally involves possibilities for various feature scales as well as link scales. A disadvantage is a heuristic nature of the criterion. One may think of theoretically modeling a community as probabilistically emerging from similar activities of its members, underlied by similarity in some individual features and external circumstances. Such a model can naturally be extended to model, for example, community evolution—an aspect that so far cannot be properly captured in the data modeling format. It would be nice to develop such a model, especially if that model would involve a criterion akin to the least squares one.

Item (ii), formulation of the criterion as a weighted sum of two squared errors, is a heuristic admission: the linear format assumes a direct interpretation of the weights as importance scores of the two data sources. Hardly one can expect this interpretation to have any operational meaning, leading to uncertainty in choosing their values. That may be considered a serious limitation of the model. Indeed, there is no reasoning regarding the data source weights except that they should balance the relative importance of the data sources. However, there could be a potential development oriented at automatic determination of the weights. Indeed, one can imagine that the least squares principle applies to the sum of the data models simultaneously rather than to each of them individually. Then the two weights can be found in an iterative process akin to that developed in [4] to determine relative feature weights for clustering.

With respect to item (iii), cluster structures, the model can be extended to overlapping clusters, but this may involve additional constraints, as pointed out in [53]. However, the possibility of extending this to hierarchical cluster structures is yet an unexplored terrain.

Last, not least, let us turn to the issue (iv) of computational complexity of the criterion. We pursue here a local search double-greedy strategy, which brings us obvious drawbacks: one cannot warrant achieving the global minimum nor even can claim any estimate of the depth of the local minimum found. Yet, there is an unexpected advantage, too: the number of communities is determined automatically in the process, not defined priorly.

What is said above leads us to list some properties which distinguish our approach from many others.

Desirable properties: a) both quantitative and categorical features are admitted; b) no restriction on the network data type; c) determining the number of clusters/communities automatically; d) a Pythagorean decomposition of the combined data scatter in the sum of contributions of individual clusters and the minimized criterion.

Less desirable properties: e) the data standardization is a necessary part of the method, both for network data and for feature/similarity data; f) slow computations; g) no advice regarding the constants balancing the relative contributions of two data sources, the network and features.

It appears that ICESi methods can be competitive indeed. On our real-world dataset collection, they win in the majority cases over state-of-the-art methods, including in the nonsummability mode. At the networks with categorical features, they show rather good performance by very closely following the winner, SEFNAC [34], and even outperforming that at some data configurations (in ss and sn modes). At synthetic networks with quantitative features ICESisn obtains best results, with occasional interventions of ICESiss.

The discussion above suggests a number of directions for future work. First of all, we should concentrate on (a) accelerating the computational speed of the method and (b) scrutinizing the balance between the network data and the feature data, so that changing the currently equal values of the balancing constants may become needed indeed.
